# Coronary Arteriovenous Fistula Originating From the Left Main Coronary Artery

**DOI:** 10.7759/cureus.24824

**Published:** 2022-05-08

**Authors:** Dilpat Kumar, Pramod Kumar Ponna, Jose R Po, Ryan Jamoua, Jagadeesh K Kalavakunta

**Affiliations:** 1 Internal Medicine, Ascension Borgess Hospital, Kalamazoo, USA; 2 Internal Medicine, Louisiana State University Health Sciences Center, Shreveport, USA; 3 Cardiology, Ascension Borgess Hospital, Kalamazoo, USA

**Keywords:** left main coronary artery, percutaneous approach, surgical management, rare clinical entity, anatomical abnormality, coronary artery fistula (caf)

## Abstract

We report a case of coronary artery fistula arising from the left main coronary artery in a 62-year-old patient presenting with atrial fibrillation. He underwent a transthoracic echocardiogram which suggested a possible coronary artery fistula. Cardiac computed tomographic angiography and cardiac catheterization confirmed the diagnosis. Coronary artery fistula originated from the left main coronary artery, which is rare and terminated in the coronary sinus. Multi-modality imaging helps to delineate anatomy and decide treatment options. Small asymptomatic fistulas do not require treatment, and large or symptomatic fistulas need closure. Our patient was asymptomatic, and we opted for conservative management with close outpatient echocardiographic monitoring.

## Introduction

Coronary artery fistula (CAF) can be congenital or acquired. They are predominantly congenital, but acquired causes are becoming more common due to the increasing incidence of coronary interventions. CAFs should be suspected when there is a cardiac murmur, an atrial or ventricular left-to-right shunt, and a large tortuous coronary artery. Larger CAFs cause symptoms due to the coronary steal phenomenon. CAFs can also cause conduction abnormalities, such as atrial fibrillation and ventricular tachyarrhythmia. The most common complication of CAFs terminating in the coronary sinus is congestive heart failure. Fistula draining into the coronary sinus is the sole anatomic risk factor for post-repair adverse events such as coronary thrombosis [1].

## Case presentation

**Figure 1:**
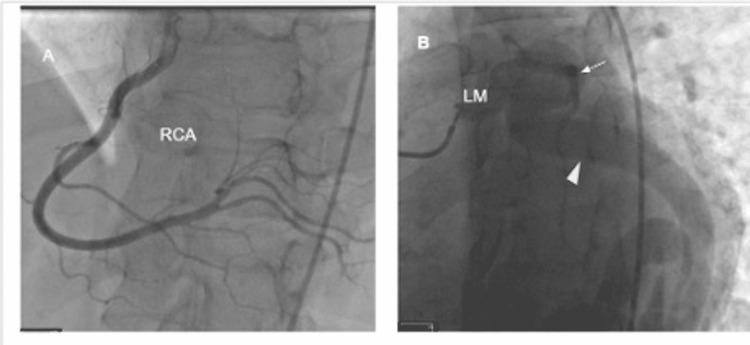
Cardiac catheterization showing A) Normal right coronary artery (RCA) in the Left anterior oblique/cranial projection B) Left anterior oblique/caudal projection showing dilated left main (LM) and dilated/tortuous left circumflex artery to the coronary sinus fistula (arrowhead) along with patent left anterior descending artery (arrow).

**Figure 2:**
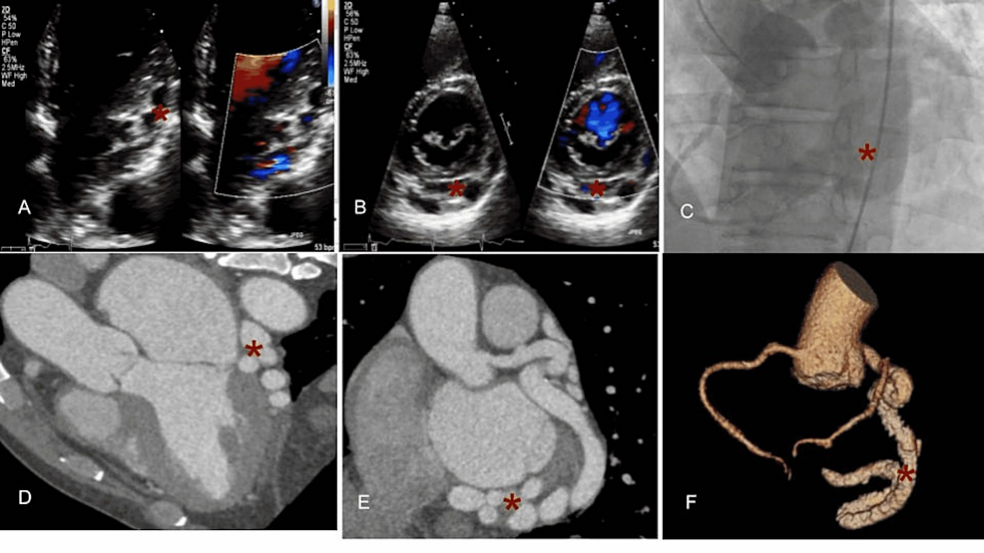
Multi-imaging modalities showing the coronary arteriovenous fistula [red asterisk*] Panel A:  Transthoracic echocardiogram apical four-chamber view showing dilated sinuses in the atrioventricular groove Panel B:  Transthoracic echocardiogram parasternal short axis view showing dilated sinuses along the inferior/inferolateral walls Panel C:  Aortic root angiogram filling the right and left coronary arterial system Panel D:  Computed tomography angiography of the heart showing sinus-like appearance and tortuous vessels in the atrioventricular groove Panel E:  Computed tomography angiography of the heart showing sinus-like appearance and tortuous vessels along inferior/inferolateral walls Panel F:  Computed tomography angiography of the heart 3D rendering showing the corresponding right and left coronary arteries along with dilated left circumflex artery arteriovenous fistula

A 62-year-old male with no significant medical history presented to our clinic after a new diagnosis of atrial fibrillation. He had no associated symptoms and endorsed excellent exercise tolerance. A transthoracic echocardiogram (TTE) was ordered, and he was started on metoprolol 25 milligrams two times daily. TTE demonstrated normal global systolic function with severely dilated right and left atrium. It also showed an ectatic left main coronary artery (LMCA) with multiple dilated sinuses along the basal and inferolateral portion of the left ventricle. Cardiac computed tomographic angiography (CTA) revealed dilated LMCA and left circumflex artery terminating into the dilated coronary sinus (Figure 1). The left anterior descending artery (LAD) is normal in size without any CAF. Subsequently, cardiac catheterization confirmed a serpiginous and aneurysmal coronary arteriovenous fistula (CAVF) communicating dilated LMCA and circumflex artery with coronary sinus, measuring 1.5 centimeters with pulmonic-to-systemic flow ratio (Qp: QS) of 1.8 (Figure 2). The patient was referred to cardiothoracic surgery for further evaluation. The discussion was performed with the heart team about various treatment options, including surgical and percutaneous closure. The decision was made to manage conservatively and watchfully, waiting for worsening clinical symptoms with a low intervention threshold.

## Discussion

CAFs account for 0.4% of all cardiac malformations and are present in 0.002% of the general population [2,3]. CAF is characterized by an abnormal conduit from a coronary artery to the cardiac chamber or great vessels adjacent to the heart [4]. In most cases, it involves the right coronary artery or LAD artery. Rarely both coronary arteries can be CAFs [5].

Our case is unique such that the origin of CAF was LMCA, which is reported in fewer than 5% of cases [6]. Most patients with CAFs are asymptomatic, but they can present with angina pectoris from myocardial ischemia due to coronary steal syndrome, exertional dyspnea, orthopnea, endocarditis in the fistula, syncope, palpitations, myocardial ischemia/infarction, or extremity swelling from right ventricular overload from left to right shunting. It has also been associated with paroxysmal atrial fibrillation, ventricular arrhythmias, and sudden cardiac death, particularly in young athletes [7]. It should be suspected in patients with unexplained heart failure or a history of chest trauma, irradiation, myocardial infarction, or cardiac procedures. Coronary angiography is the primary diagnostic and therapeutic modality for assessing CAF. Non-invasive modalities such as multidetector cardiac CTA and magnetic resonance imaging can also provide detailed information regarding the anatomy, including origin, patency, and termination of CAF [8]. Non-invasive imaging modalities facilitate the diagnosis and are used as an adjunct to coronary angiography. Selective coronary angiography is required to accurately assess fistulous tract anatomical features and cardiac hemodynamics and identify other structural abnormalities [6]. The American College of Cardiology (ACC) and American Heart Association (AHA) recommends monitoring small asymptomatic fistulas while closing fistulas greater than 250 millimeters irrespective of symptoms and all symptomatic fistulas regardless of the size [9]. Closure can be achieved with a percutaneous transcatheter approach or surgically with sternotomy. Surgical closure is recommended in patients with symptomatic or asymptomatic CAF with other cardiac pathology or unfavorable vascular anatomy. Transcatheter closure is recommended in patients with symptomatic or asymptomatic CAF and suitable vascular anatomy [10,11].

Surgical closure is the gold standard for CAF treatment with good reported success, safety, and efficacy. More randomized multicenter studies are required to evaluate long-term survival and effectiveness of surgical versus transcatheter closure. Patients with proximal, non-tortuous, and easily accessible fistulas are the candidates for occlusion with coils and plugs with an angiography [9]. Guidelines recommend following small, asymptomatic fistulas every 3-5 years with repeat, non-invasive modalities to monitor the progression [9]. Pharmacological management includes anti-anginal therapy, antiplatelet therapy, and standard endocarditis prophylaxis [12].

## Conclusions

CAF is a rare congenital heart disease, mainly diagnosed incidentally on echocardiography or angiography. Coronary arteriovenous fistula rarely arises from the left main coronary artery. CAFs can present with angina pectoris, exertional dyspnea, paroxysmal atrial fibrillation, ventricular arrhythmias, or sudden cardiac death. ACC and AHA recommend monitoring small, asymptomatic CAFs while closing larger fistulas or those causing symptoms/hemodynamic instability. Proximal non-tortuous CAFs should be managed with angiographic coiling and plugging. Depending on vascular anatomy and associated comorbid conditions, a percutaneous or surgical approach can be utilized.
